# Topological Characterization of Metal–Organic Frameworks: A Perspective

**DOI:** 10.1021/acs.chemmater.4c00762

**Published:** 2024-07-22

**Authors:** Lawson T. Glasby, Joan L. Cordiner, Jason C. Cole, Peyman Z. Moghadam

**Affiliations:** † Department of Chemical and Biological Engineering, The University of Sheffield, Sheffield S1 3JD, United Kingdom; ‡ Cambridge Crystallographic Data Centre, Cambridge CB2 1EZ, United Kingdom; § Department of Chemical Engineering, University College London, London WC1E 7JE, United Kingdom

## Abstract

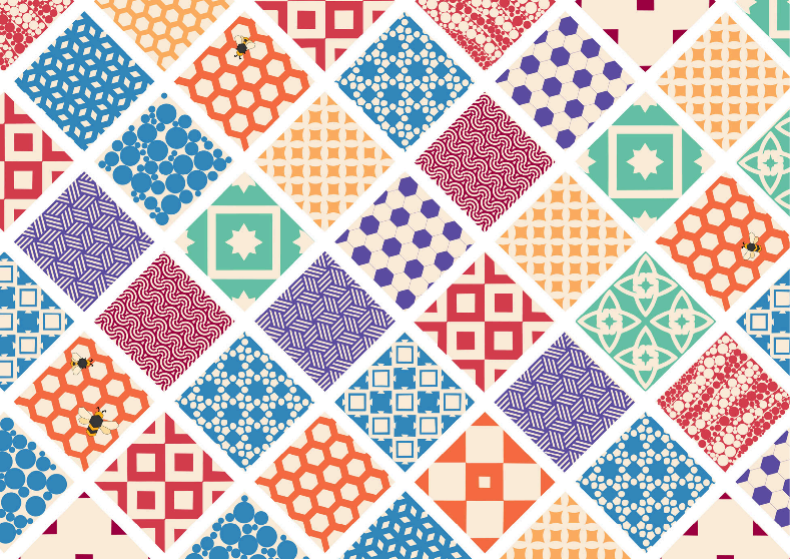

Metal–organic frameworks (MOFs) began to emerge over two decades ago, resulting in the deposition of 120 000 MOF-like structures (and counting) into the Cambridge Structural Database (CSD). Topological analysis is a critical step toward understanding periodic MOF materials, offering insight into the design and synthesis of these crystals via the simplification of connectivity imposed on the complete chemical structure. While some of the most prevalent topologies, such as face-centered cubic (**fcu**), square lattice (**sql**), and diamond (**dia**), are simple and can be easily assigned to structures, MOFs that are built from complex building blocks, with multiple nodes of different symmetry, result in difficult to characterize topological configurations. In these complex structures, representations can easily diverge where the definition of nodes and linkers are blurred, especially for cases where they are not immediately obvious in chemical terms. Currently, researchers have the option to use software such as ToposPro, MOFid, and CrystalNets to aid in the assignment of topology descriptors to new and existing MOFs. These software packages are readily available and are frequently used to simplify original MOF structures into their basic connectivity representations before algorithmically matching these condensed representations to a database of underlying mathematical nets. These approaches often require the use of in-built bond assignment algorithms alongside the simplification and matching rules. In this Perspective, we discuss the importance of topology within the field of MOFs, the methods and techniques implemented by these software packages, and their availability and limitations and review their uptake within the MOF community.

## 1 Introduction

Metal–organic frameworks (MOFs) are an emerging class of porous materials, formed by chemical bonds between metal clusters and organic building blocks.^1,2^ MOFs are a diverse set of chemical structures often characterized by their porosity and customizability: the commercial uptake of MOFs are particularly focused toward gas adsorption,^3,4^ separation,^5−7^ sensing,^8,9^ alongside catalysis^10,11^ and quantum applications.^12−15^ The MOF materials space consists of many combinations of building units typically configured in a symmetrical pattern. These building units are often referred to as Secondary Building Units (SBUs). SBUs are the fundamental components of the framework, typically consisting of metal ions or clusters and organic linkers that combine to form the periodic structure of the MOF. The precise nature and arrangement of SBUs within a MOF determine its structural and functional properties. Over time, increased importance has been placed on topology as a predictor of properties: recently investigations have been published that compare topology with porosity and mechanical stability,^16,17^ but there are still areas in which potential correlations between topology and other properties have not been determined, such as electronic properties, solvent compatibility, and thermal stability.^18^

**Figure 1:**
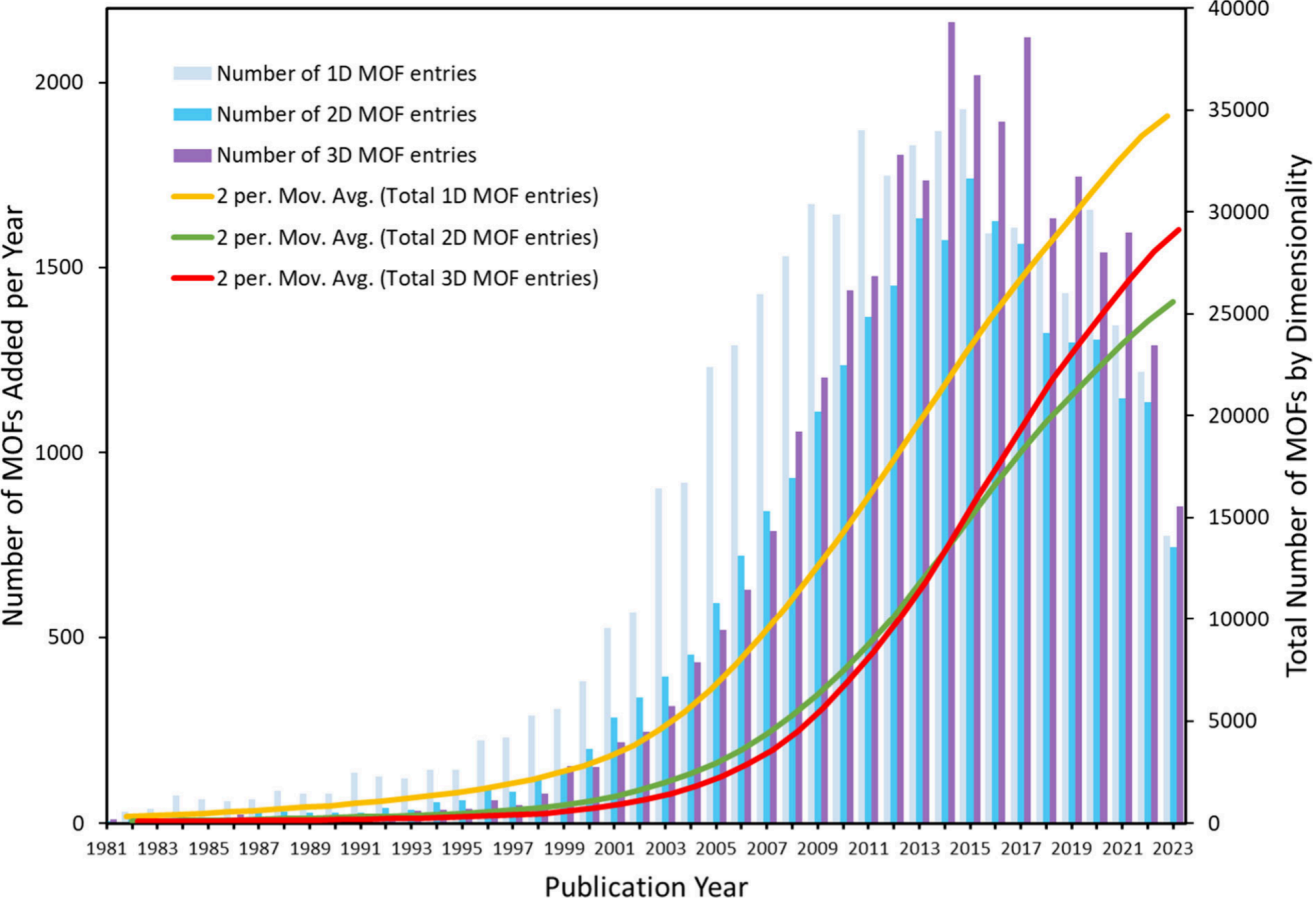
Distribution of MOFs within the CSD, including dimensionality breakdowns of 1D, 2D, and 3D structures. The left axis indicates the number of structures deposited per year per dimensionality, while the right axis keeps a cumulative total across the timeline. (Data correct to CSD 5.45 November 2023).

The CSD MOF subset contains a staggering ca. 120,000 experimental crystal structures of MOFs (CSD release April 2023), representative of the input of the worldwide research community, with updates to the total number of synthesized structures being made quarterly.^19−21^Figure 1 shows the distribution of MOFs within the CSD from 1981 to present day, including a breakdown of their structural dimensionalities. While there appears to have been a clear preference toward the synthesis of 1D MOF-like structures from the inception of the CSD until 2011, there has been a recent increase in the popularity of 3D structures compared to the initial high proportion of 1D deposits. The initial prevalence of 1D MOFs could be explained by the cost-effective formation of simple structures consisting of basic pyridyl and chelate ligands, typically synthesized with the intention to study these ligands and their interactions with metal centers. These 1D chains have interesting applications in magnetism, proton conductivity, and ferroelectricity and can often form larger crystals than equivalent 2D and 3D structures under ambient conditions. We note that, despite their dimensionality, these structures can exhibit porosity when linked by hydrogen bonds or other interactions, when woven together/interpenetrating (1D+1D), or they could potentially exhibit porosity on desolvation.^22^ 3D MOFs are typically considered to be the ideal candidates for adsorption applications and the increasing focus on 3D MOFs can be seen in the cumulative 3D structure deposits (red line in Figure 1) where they begin to overtake 2D submissions in 2015. The number of 3D MOF submissions to the CSD has consistently exceeded 1000 accepted annual deposits for the last 15 years.

Following the International Union for Pure and Applied Chemistry (IUPAC) recommendations, published in 2013, suggesting that all MOF structures are assigned topological representations, a significant number of these materials should now be published and deposited with accurate topological information.^23^ Ohrstrom et al.^24^ released an informative review in 2015 following the publication of these IUPAC recommendations, where they offered guidance to researchers working in the field of MOFs surrounding identification of nets and network topologies. At present, the CSD does not report network topologies of its deposited structures, although for many materials submitted since 2013, this information may be available within the corresponding manuscripts as evidenced by our previous study which included the text-mining of MOF topologies.^25^ The suggested procedure for reporting MOF network topologies is using a unique three letter code taken from the Reticular Chemistry Structure Resource (RCSR), printed in bold lowercase letters.^2^ The RCSR is an open source, online database consisting of 2,929 3-periodic, and 200 2-periodic network representations. It is self-described as a collection of spatial information, and corresponding diagrams, which can be used to map networks that are built using straight, nonintersecting linkers.

Additional alternative databases for topological descriptions do exist, these primarily include the Topological Types Database (TTD)^26^ and Euclidean Patterns In Non-Euclidean Tilings (EPINET)^27^ theoretical database. While there is often some overlap between these collections, it is very common to see newly reported structures represented in literature by their corresponding RCSR identifiers. Where the RCSR representation is not present and if the topology has been determined by the authors, the alternative EPINET or TTD terminology may be seen. Typically, topological identification software packages refer to the RCSR labels with a preference over other representations wherever it is possible to do so, although RCSR and EPINET topologies are sometimes reported together. It is worth noting that RCSR topologies appear in the EPINET database with a different unique reference, for example the RCSR **pcu** is also represented by the EPINET s-net name sqc1, and likewise **bcu** can be reported as sqc3.

As the CSD does not contain topological information, and there is at present no publicly available complete MOF topology database, to obtain the topology for a given MOF structure one would need to search for the corresponding topology in the respective publication, or if this was not available, determine the topology for the structure by using one of the existing software packages. This article discusses the use of three readily available MOF topology identification programmes: Topos Pro,^26^ hosted by Blatov and colleagues from the Samara Topological Data Centre, MOFid^28^ published by the Snurr Group at Northwestern University, and finally CrystalNets^29^ a Julia based software from Chimie ParisTech published by the Coudert lab. Each of these approaches differ, sometimes subtly, in the structure connectivity, deconstruction, and identification stages. We also explain the important challenge of bond assignment and different approaches to topological identification and compare different software features that are currently available. We also discuss the techniques used to obtain deconstructed or underlying nets, and current examples of data sets created using these packages.

## 2 What Is Topology?

A long-recognized feature of crystal chemistry is that the connectivity between atoms can be represented as a simple periodic graph. This is particularly evident in metal–organic frameworks (MOFs), where linkers act as edges and metal ions or clusters serve as nodes, allowing these atomic arrangements to be simplified into graph structures. Covalent organic frameworks (COFs), zeolites or any other periodic crystal structure can similarly be represented in this way. If they do not contain metals; any atom with a connectivity greater than two is considered a node. Topology, the mathematical study of spatial properties preserved under continuous transformations, plays a crucial role in structure analysis. Famously summarized by A.F Wells in his 1977 book on Three-dimensional Nets and Polyhedra,^30^ topological analysis provides deeper understanding of crystal materials and their properties, enabling comparisons of new materials with existing literature, and effectively communicating the networks of new materials. Other important concepts of topological representation include homeomorphisms, fundamental groups, and homology groups, particularly when investigating materials for their porosity characteristics.

Topology holds significance beyond the simplest natural structures such as diamonds, zeolites, and quartz to describe and understand the variety of crystalline materials. Even in these simple one atom type configurations, the structural connectivity at atomic scale can affect the properties of the macrostructure. If we consider only carbon, while diamond, with its instantly recognizable cubic lattice construction registers at the peak of the hardness scale, lonsdaleite is built using a hexagonal lattice configuration and is potentially up to 58% harder than its cubic counterpart when measured across the <100> face.^31^

In 2019, Moghadam et al.^17^ reported the correlation between structure-mechanical stability and topology for 3,385 MOFs and 41 distinct topologies. In this context, they identified the top robust network topologies and emphasized the importance of building blocks, coordination numbers, and linker lengths. Later, in 2022, Li et al.^32^ experimented with different synthesis conditions and concluded that it is possible to control the formation of specific topologies for a set of identical building blocks which can be useful to consider if a certain pore shape, size, or stability is desirable. The formation of distinct MOF nets from the same building blocks is an important insight into consider as it demonstrates the remarkable structural diversity and flexibility of MOFs and underlines the importance of the principles of MOF formation.

In 2018, Bonneau et al.^33^ published terminology guidelines to aid in the deconstruction of crystalline networks into their underlying nets. Their estimation suggested that 40,000 MOFs would be synthesized and published by 2025, a result that seems almost achievable given the 28,729 3D MOFs offered in the CSD release of April 2023, or one that already has been achieved if we include 2D MOFs within the prediction. One important focus of these guidelines was to address the ambiguity of node assignment. The method through which the nodes are chosen can have a significant impact on the outcome of topological assignment, depending on the constituent building blocks. If, for example, large linkers with porphyrin rings are present, the style of deconstruction approach can offer different outcomes to the most basic structure form. The general goal is to represent the connectivity of a structure using an underlying net which is mathematically defined as a simple periodic graph, consisting of vertices and edges. A simple graph is made suitable for modeling topological representations of MOFs by four important criteria:

1. Edges are nondirectional, only a Boolean result when questioning connectivity between two nodes is required.
2. Nodes cannot exist which have only 1-connection, they must be considered “loose ends” and removed. Elements such as hydrogen cannot become nodes.
3. A node cannot be connected to itself, there are no loops, and although this is not expected when approaching MOFs, it must be considered.
4. Each node connects only once to another node, additional connections between two of the same nodes are discarded. In some instances, where for example a MOF has a double linker between two nodes,^34^ these must be simplified into a single edge.

A net must be connected, periodic, and simple; this is the minimum information required to construct a good topological representation. Where MOFs and other periodic structures are concerned, periodic boundary conditions (PBCs) are employed to simulate infinite lattices by repeating the unit cell in all spatial directions. This approach is essential for accurately modeling the bulk properties of materials and eliminating edge effects. When applying PBCs, attention is required to handle the connectivity of nodes at the boundaries of the unit cell. We define above that nodes cannot be connected to themselves within the finite cell, however, under PBCs, a node at one boundary is effectively connected to its periodic image at the opposite boundary, creating a seamless, infinite network. These boundary connections should not be misconstrued as self-connections, as they result from the periodic repetition of the cell.

**Figure 2:**
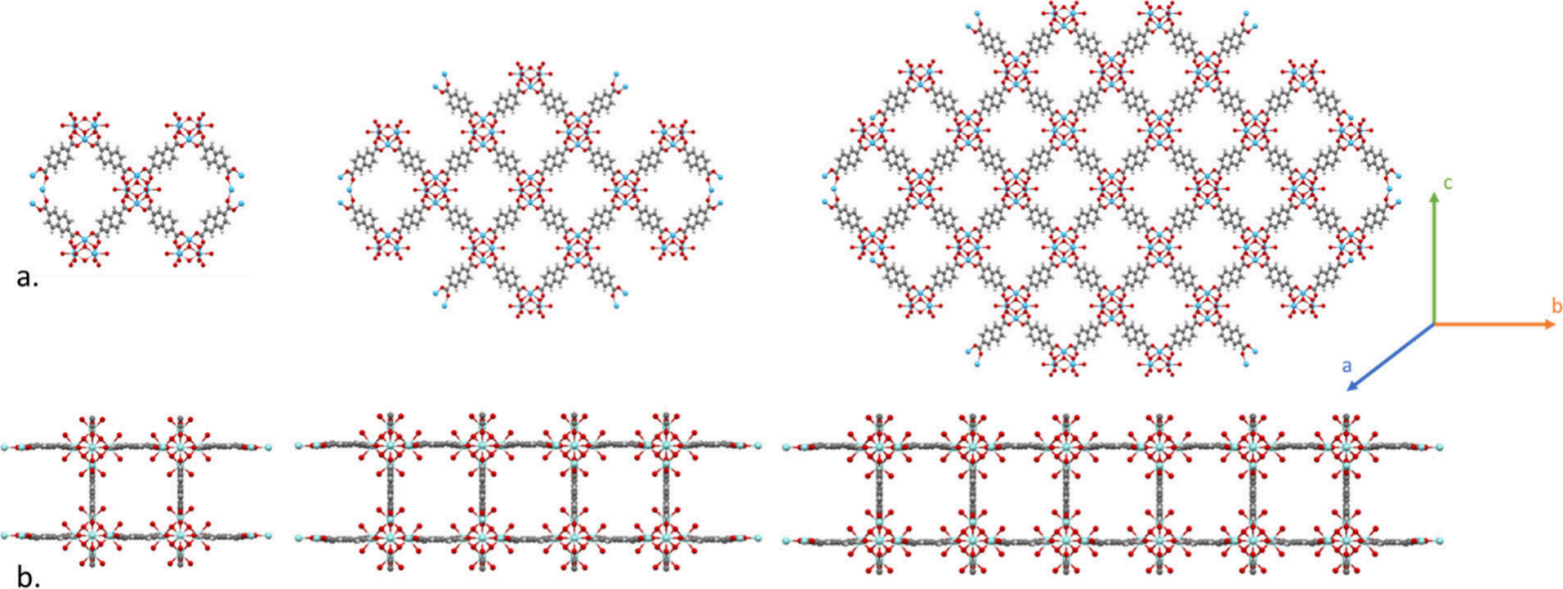
An example of two similarly connected crystal structures expanded 1× , 2× , and 3× from their unit cells, where (a) CSD OFAWAV (DUT-53(Hf)) consists of 8-connected SBUs and (b) CSD OFAWID (DUT-84(Zr)) consists of 6-connected SBUs, visualized using CCDC’s Mercury.^35,36^ The latter entry is considered disjoint due to the lack of polymeric expansion sites along the *c*-plane; however, it expands polymerically in both other planes. Hf (bright blue), Zr (cyan), O (red), H (white), and C (gray).

Topology can be represented for any periodic crystal structure in both 2D and 3D planes, and for both cases the same rules apply. Structures that are 3D but only “grow” into two planes (2-periodic) are known as *disjoint*, and do not have a true topological representation when considering RCSR criteria, although some representations for these types of crystal can be found in the TTD. Figure 2a. demonstrates the 3-periodic **bcu** topology CSD OFAWAV (DUT-53(Hf)) structure expanding polymerically from its 8-connected SBU in all 3 planes of space, yet Figure 2b. shows the existence of “stunted” nodes on CSD OFAWID (DUT-84(Zr)), a derivative of the **bcu** based structure, where we see expansion in only two of the possible three planes originating from the now 6-connected SBU.^35^ Here, two atomic scale sheets have been layered and are bonded by a linker, but in this case, there is no potential for expansion via further bonded sheets in the **c** plane for this structure, and therefore any subsequent layers would be treated as separate structures, like stacking sheets of corrugated cardboard. For this structure, the disjoint configuration is due to the deliberate replacement of linker molecules on the 8-connected SBU metal clusters with acetic acid molecules, resulting in a 6-connected SBU leading to a restricted 2D structure consisting of double layers. Interestingly, the pore limiting diameter (PLD), and the maximum pore diameter are not drastically changed between each configuration, and when shifting from **bcu** to the disjoint structure we see them reducing from 8.5 to7.6 Å, and 11.2 Å to 11.1 Å, respectively.^35^ As a result, we might expect to find several deliberately disjointed structures within the CSD’s 2D MOF subset that demonstrate a comparable level of porosity to 3D structures.

Clearly, there is a requirement for a rigorous and well-defined way to describe the symmetry demonstrated in MOFs, which could be extended to other crystal structures that consist of repeating units. This is generally accepted to be best represented by repeating the structure according to one of the 230 space groups found in the International Table for Crystallography Volume A.^37^ After the space group of a structure has been determined, it is typically followed by the allocation of coordinates for each unique metal node in a unit cell, designed to create an infinitely expandable 2D or 3D network representation of a structure where there is little room for ambiguity.

The next, and truly key, step in the topology identification process is defining the positions of atoms that make up the nodes and linkers of the structure. Once coordinates are assigned to a vertex it is then designated as a node and the same applies to edges and their distinction as linkers. Although coordinates may be assigned by a variety of methods, the topology can be identical for structures that have different geometry. The creation of several nets may lead to a group of isomorphic representations, although it is often recommended that the network with the highest symmetry should (in these cases) be chosen as the universal net. This is somewhat subjective as it is often the whim of the crystallographer that decides the outcome as there are currently no set rules or absolutes for topological assignment, and it appears likely that will remain the case for the foreseeable future. There are several valuable discussions available for further reading that focus on the assignment of topology based on metal–organic polyhedra, such as the contributions from Goesten et al. in 2013^38^ followed by Kim et al. in 2015.^39^

Additionally, our discussion here must mention the existence of interpenetrating structures in which the empty space between nodes may accommodate one or more additional networks. While the description and relationship between two 3D nets is quite straightforward, the complexity of possible relations between 2D sheets, or 1D chains, is significantly increased.^40−42^ Interpenetrating MOFs, often referred to as IMOFs, can display some fascinating topologies and architectures and they often exhibit improved functions for certain applications. The existence of homo- and hetero- IMOFs can make for interesting discussion surrounding the topology of these structures and the representations that are allocated to them, particularly those created using two or more underlying structures that results in a change of dimensionality for the macroscale material. Typically, each separate structure is considered during topological assignment rather than considering the interpenetrating nets as a single material, IMOFs do not contain bonds between the nets that are interpenetrated as they typically form independent structures inside the pores of each other. As an example, some MOFs can consist of many layers of the same 2D sheets interpenetrated throughout the entire structure to give an infinite number of 2D sheets where only one topological assignment needs to be made. An identical procedure is followed where these simplified nets are then matched to pre-existing representations found within the RCSR. We note that the interpretability of topology can also create barriers toward having exact solutions for each structure where additional representations are arguably equally suitable for an underlying representation.

## 3 Popular Topologies and Resources

### 3.1 The Reticular Chemistry Structure Resource (RSCR)

The RCSR was developed as a database to aid in both the design of new structures and the analysis of existing structures.^2^ The latter being particularly useful as a considerable number of materials in the CSD were deposited before the popularity of MOFs began to boom, and in fact before the distinction of these structures was made in the early 2000s.

The RCSR consists of four sections, 0-, 1-, 2-, and 3-periodic nets. These are also split into two subsections of default or woven nets. Woven nets contain tangled polyhedra, chains, interlocked components, weaving and interpenetrating nets, and multicomponent structures. For the default setting, the 0- periodic set contains structures consisting of convex polyhedra, including cages with 2-coordinated vertices. The 1-periodic list consists of cylindrical tilings and unsurprisingly, the 2-periodic set consists of plane tilings. Finally, the bulk of the RCSR, and the most interesting collection for those with an interest in gas adsorption, separation, and other porous applications of MOFs, is the 3-periodic set containing embeddings of periodic graphs. These structure definitions have been collected over a period from 2003 to present day in a series of important works.^43−51^

**Figure 3:**
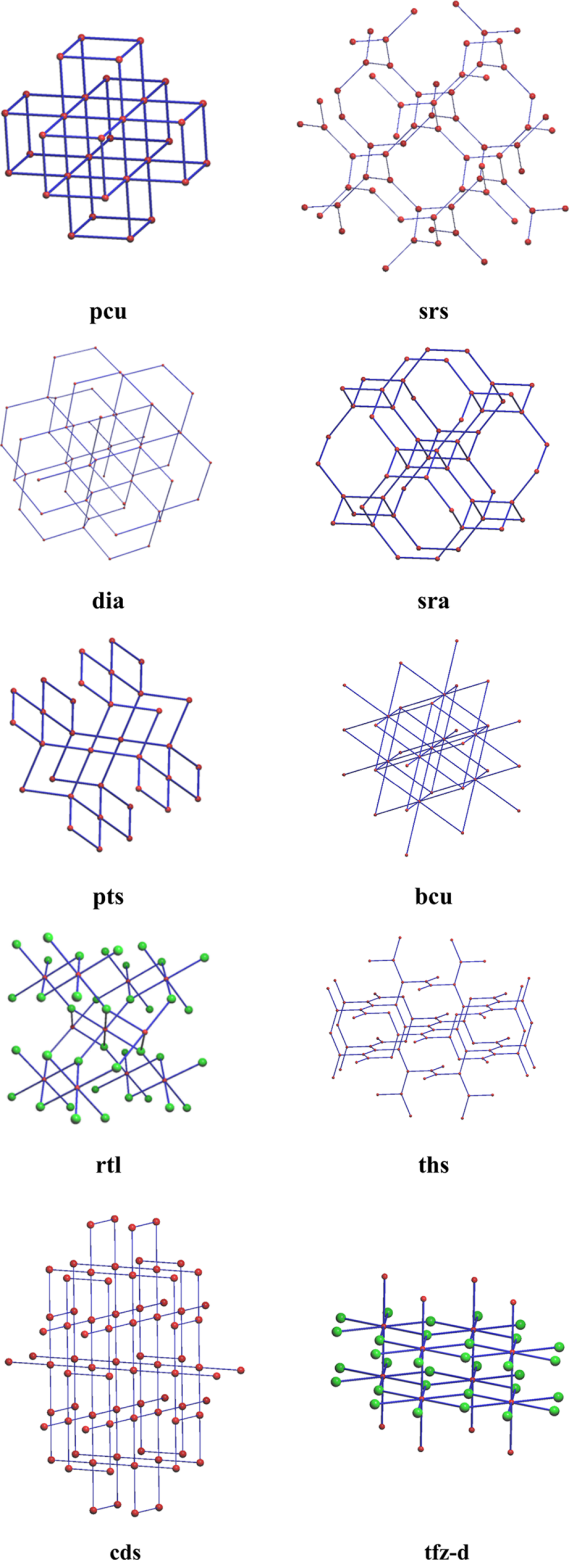
Example RCSR topological nets created and visualized using ToposPro.^26^ Red atoms represent metal nodes, whereas green atoms represent organic nodes.

In the RCSR, each topology is given a unique 3-letter identifier, typically reported in bold. These are sometimes presented with a simple suffix providing additional information. Each entry contains information regarding the vertices and their symmetry, coordinates, coordination, and order, with the same provided for edges, besides coordination. It is this data which is necessary to match these representations to simplified MOF structures, and these representations that are often reported in catalogues of MOF data. Figure 3 shows a collection of 10 of the most commonly occurring 3-periodic RCSR nets found in the CSD 3D MOF subset.^25^

In this section, it is again worth mentioning the TTD, and the EPINET resource as other examples of topological collections that are notably relevant to the underlying connectivity of MOF structures. However, due to the limited availability of the TTD database without a license, we focus our discussion on the RCSR collection. This is solely to ensure that fair comparison can be made between the topology assignment software packages detailed in Section 4. For completeness, all the structure representations in Figure 3. can also be found in the EPINET collection by searching the related RCSR names to find the corresponding sqc’xxx’ style reference codes.

Here, we point out the existence of zeolite framework type descriptors that are also represented by 3 letter reference codes.^52^ These are an older resource than MOF topologies with rules on nomenclature dating back to 1979,^53^ and are unrelated to the RCSR. The 3-letter codes are typically derived from the material or institution origins, for example faujasite becomes FAU, and a complete list can be viewed here https://europe.iza-structure.org/IZA-SC/Zeolite_names.html. However, this is not to say that RCSR topologies could not be assigned to zeolites, and the use of capitalisation should set them clearly apart from lowercase RCSR references.

Lastly, the use of the RCSR is not restricted only to MOFs and zeolites. It can be applied to any crystal structures including covalent organic frameworks (COFs), AlPO and GaPO structures, or even formations of single atom lattices that would match with the RCSR’s structure descriptors.

### 3.2 Edge-Transitive Nets

Transitivity is a concept that describes symmetry and uniformity of nets based on how the vertices, edges, and faces can be mapped through net symmetries. Low transitivity is often correlated with high symmetry which can impact the physical properties of a crystal, and therefore dictate its potential applications.^48^ Vertex, edge, and face transitivity are all important considerations, and understanding a structures transitivity can offer insight into the predictability of a materials behavior.

While edge-transitive nets are often reported for many MOF structures, they may not necessarily be considered as the underlying topology of a structure. Edge-transitive nets are typically used to describe the structural symmetry, as opposed to the connectivity of the nodes and linkers. By selecting any edge in an edge-transitive net it is possible to rotate or reflect the structure around that edge and observe the arrangement of linkers and nodes remains unchanged. The nets represent a particular structure symmetry and can be used to design and synthesize MOFs with specific properties.

On the contrary, underlying nets are not restricted by the specific arrangement of linkers and represent only the spatial arrangements of nodes and connections. Edge-transitive nets are typically derived from the underlying nets, for example the underlying basic **nts** net can be obtained from simplifying further a derived net **ntt** structure. The derivations often consist of assigning geometric polyhedra to the nodes, and across some linkers, to have further influence on the exact shapes that can be obtained from a certain net. There are several ways in which one net may be considered a derivative of another, these include subgraph construction and coordination changes, topological transformations such as framework augmentation i.e insertion of new SBUs, or by increasing/reducing dimensions.

Chen et al.^54,55^ have worked on reviewing minimal edge-transitive nets specifically for the design and development of MOFs, and Hoffmann’s Introduction to Crystallography^56^ discusses details surrounding the basic and derived nets found in the RCSR, supplemented by an online resource.^57^ A recent contribution from Delgado-Friedrichs et al.^58^ discusses some new results and contains a concise review on 3D tilings and surfaces.

## 4 Deconstruction Techniques

Embedded within the topological identification software packages are several algorithms that are typically applied to a basic (i.e., containing no additional information such as atomic bonding) CIF to determine the simple underlying connectivity of the structure provided. Each algorithm takes a slightly different approach to simplification, and as metal nodes can be assigned subjectively, it is important to understand the differences between the techniques and how they operate. All methods first define which groups of atoms should be considered as nodes, and subsequently which connecting branches become the linkers. It is worth noting that some linkers may contain metals which are not necessarily assigned as nodes, for example in a metallic porphyrin ring (CSD BEDYEQ^59^), and conversely a linker may contain an organic ring which is best represented by a node, albeit an organic one (CSD JOZWIG^60^). It must also be considered that, for a topological representation, there is no difference between the types of nodes which exist in a simple periodic graph as there is no absolute distinction between metals and organics in these underlying representations.

The typical algorithms employed in MOF deconstruction include, all node,^61−63^ single node, standard representation,^26^ and metal-oxo.^28^ An additional cluster representation method is a partial but chemically reasonable deconstruction technique that requires the division of all bonds into intercluster and intracluster criteria. In what follows, we outline the steps performed by each of these algorithms and include schematic diagrams to aid understanding via visual representation of these stages.

### 4.1 All Node and Single Node Deconstruction

**Figure 4:**
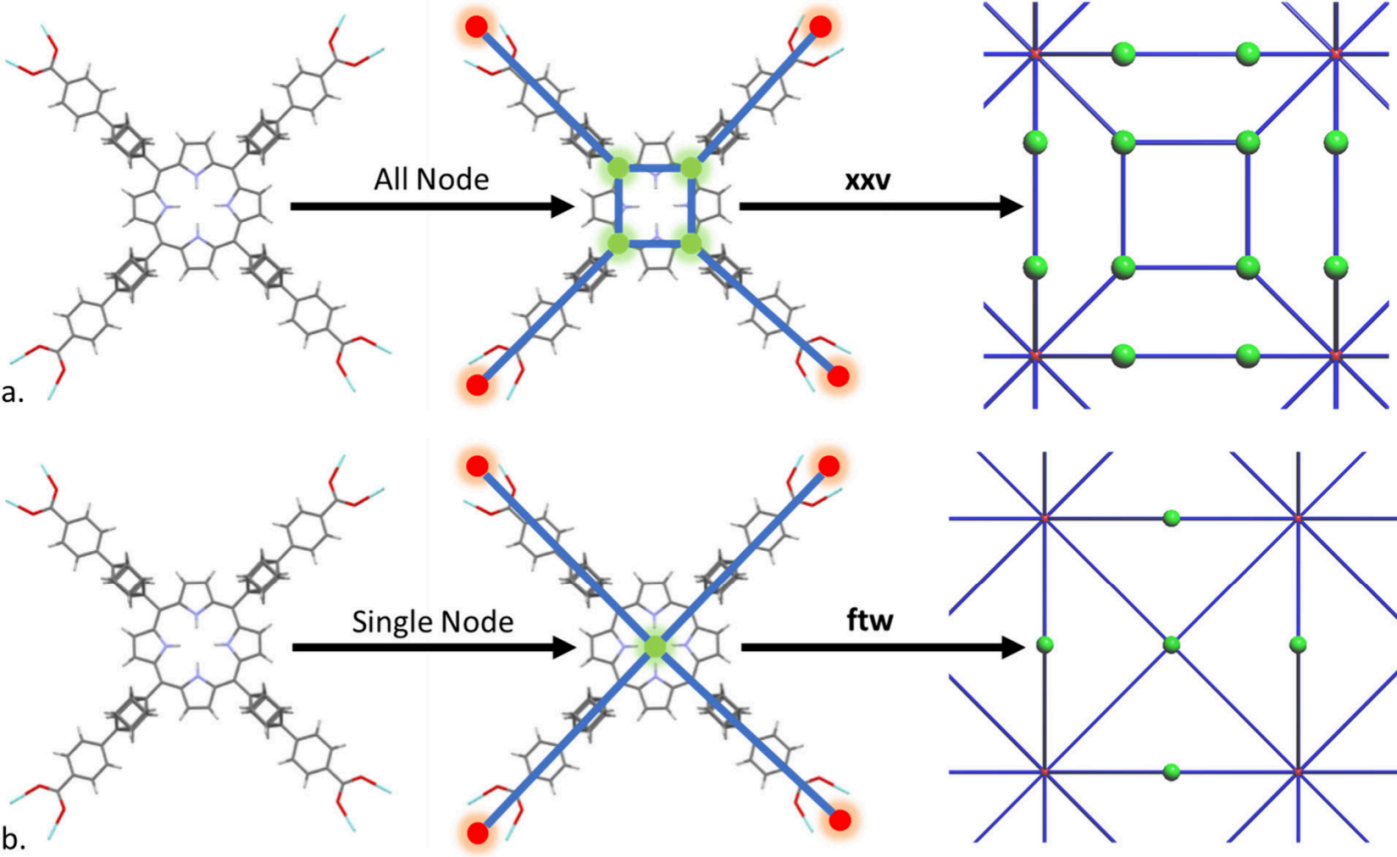
Schematic demonstrating crystal deconstruction techniques applied to CSD JOZWIG.^60^ The distinct path taken by each algorithm for large heteroaromatic rings results in (a) the all node approach matching the **xxv** topology and (b) the single node approach matching with the **ftw** topology. Wireframe structures show C (gray), O (red), N (blue), and Zr (light blue), which are simplified to metal nodes (red), and organic nodes (green) connected by straight edges representative of linkers (blue).

The most recent publication describing the all node algorithm was from the work of Li et al. in 2014.^61^ However, earlier examples have been published as far back as 2006.^62,63^ This algorithm works by considering inorganic nodes and organic linkers as abstract shapes (polygons and polyhedra) connected in a simplified net. Connected carboxylates and heteroaromatic rings are considered to constitute part of the node. After the nodes and linkers have been assigned, these clusters are simplified via replacement with pseudoatoms at geometric centers. Any isolated pseudoatoms are considered free solvents and are removed from this simplified net. Figure 4a. demonstrates the steps undertaken to assign an all node net for an atomic level crystal structure. Here, the metal clusters, formed of polygons, are treated as a single polyhedron and simplified to a single inorganic node. Similarly, the porphyrin ring is considered also to have been built with polygons, which are used to create a single polyhedron with four pseudoatom connecting points on the vertices.

**Figure 5:**
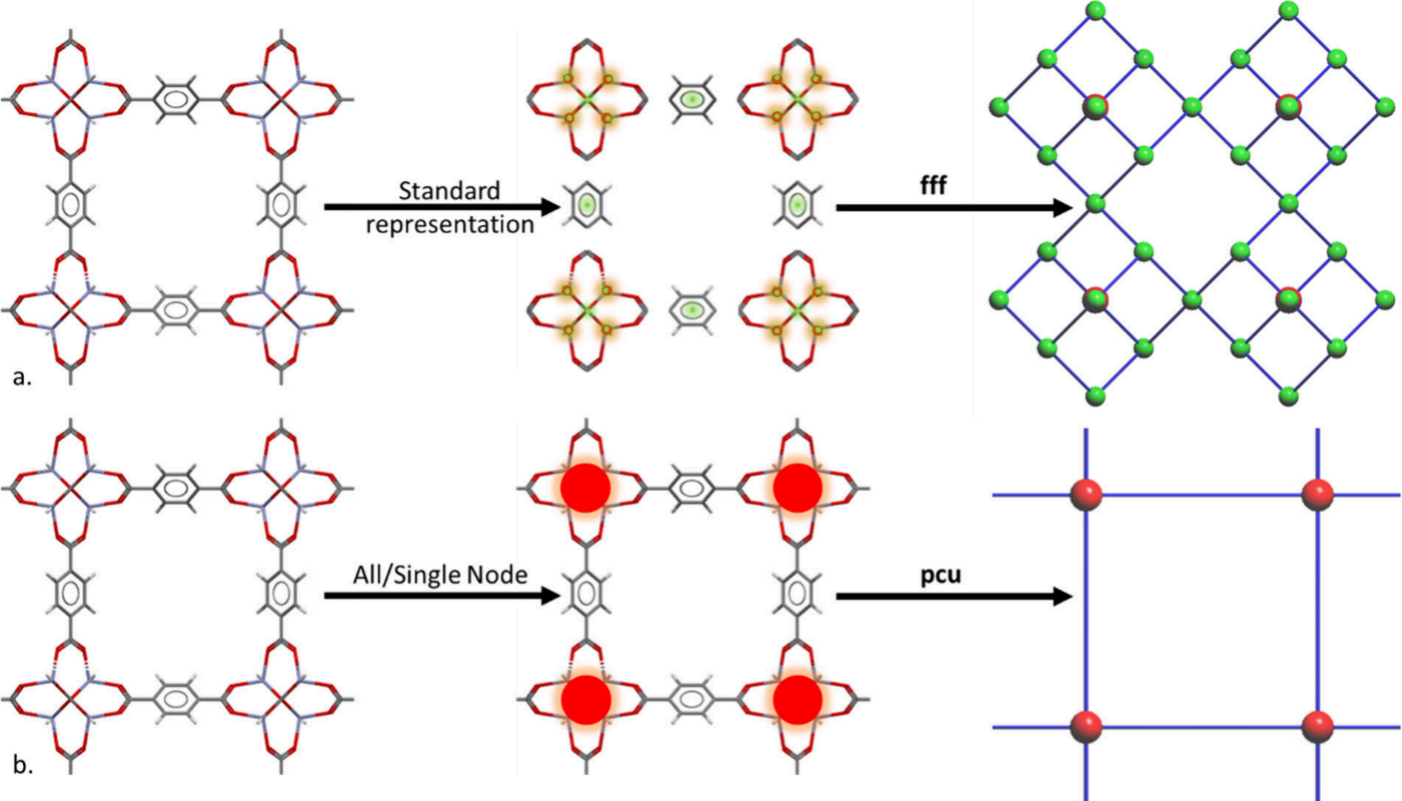
Schematic demonstrating crystal deconstruction techniques applied to CSD SAHYIK.^66^ The approach, (a) standard simplification, with initial disconnection between metal atoms and the organic structural units results in a match with **fff** topology and (b) all/single node matches with **pcu**. Wireframe structures show C (gray), O (red), and Zn (blue), which are simplified to metal nodes (red), and organic nodes (green) connected by straight edges representative of linkers (blue).

This approach specifically identifies branching points within the linkers of a MOF to provide additional information about the underlying structure, but this allows for the creation of ambiguous branching nodes. Typically, the all node algorithm creates a more complex structure which can be matched to nonparent nets in the RCSR. For example, for the structure shown in Figure 5, the **xxv** net can be considered a derivative of the **ftw** net. O’Keefe et al.^64^ explains there are many situations in which retained information takes precedence over reporting only the most simplified parent net. Using these nonparent nets can often be useful for comparing similar structures because of the retention of this important higher-level connectivity information and it makes the discovery of closely geometrically related structures much easier.

The single node approach is similar to that of the all node approach, however pseudoatoms with only one neighbor are dealt with based on their identity. Either metal containing linker molecules show up as pseudoatoms with nonredundant connections to a linker and therefore are merged, or linkers with a single connection, except for single nonoxygen atoms such as halogens, are removed as unnecessary bound solvent molecules. This approach is demonstrated in Figure 4b. where the difference between the all node algorithm above can be noted for the simplification of the large aromatic ring structure. Here, the metal clusters are treated the same way as above, but the porphyrin ring is instead considered to be a single point, rather than a polyhedron with separate vertices and edges.

The Single node approach is often considered the preferred technique to determine the most basic nets in MOF chemistry as it typically reports the parent net of structures that may also have alternative complex representations. It is anticipated that most reported topologies are obtained using the single node approach, and this allows for easier categorization of structures into broader topology groups. The allocation of **xxv** and **ftw** topologies to this same structure can both be considered correct; we must remember that one is only a more complex net that has been derived from the other. As the simplifications to the structure are only being conducted differently due to the choice of algorithm used, either representation is permitted.

Overall, the single node method describes the most basic form, whereas the all node algorithm retains complexity. It is essentially down to the preference of the researcher to determine which outcome they consider more favorable, although it is worth noting that for many materials both algorithms will report the same result as they have only one valid representation. The IUPAC recommends that researchers should report multiple topologies if appropriate, in this case when reporting the all node result, we would expect to see a statement like “the **ftw**-derived net **xxv**” which should be stated alongside the **ftw** single node outcome.^23^

### 4.2 Alternative Deconstruction Methods

#### 4.2.1 Standard Representation (Standard Simplification)

This is perhaps the simplest of all the algorithms mentioned in this list, it is concerned with disconnecting any bonds to metal atoms and leaving the remaining molecular graph intact.^65^ Standard representation is concerned only with a conventional crystallochemical description in which metal atoms and organic ligands are the only structural units, the types of bonding considered are valence only, and all atoms of each ligand are substituted by a pseudoatom. More generally, anything classed as nonmetal will be contracted to a single atom at the center of mass including but not limited to single nonmetal atoms such as oxygen, halogens, or multiatomic noncoordinated species.

For the case demonstrated in Figure 5a. where this simple technique is applied to MOF-5 (SAHYIK) from the CSD 3D MOF subset, we can see that a significant number of bonds are retained. This method is shown in parallel to the previously described all or single node approaches shown in Figure 5b., generating a distinct difference in outcome. Where standard representation here assigns a more complex **fff** topology consisting of significantly more pseudoatoms, the all or single node approach selects only the metal nodes in a more extreme simplification represented by the **pcu** topology that could be considered a loss of key information.

In addition to this approach, there is a second method detailed by Barthel et al.^65^ called cluster simplification which recognizes clusters of atoms with high connectivity. This technique draws many similarities to the all node and single node algorithms, and has been used to determine if two separately deposited structures are the same. For example, rotating a linker of a specific MOF may not change the material, but it could have an impact on the space group which in some circumstances would allow the structure to be redeposited into the same database. In this technique, the smallest ring of bonds is found for each bond. Next, the ring sizes, a, are sorted by increasing value from a_1_ to a_N_, where N is the number of bonds in the structure, in the sequence a_1_ ≤ a_2_ ≤··· a_N_. If the sequence contains a pair a_j_, a_j+1_ such that a_j_ – a_j+1_ > 2, the bonds where the smallest rings are formed by less than i+1 bonds belong to a cluster, and the others connect two clusters together. Each cluster is substituted by a pseudoatom to obtain *i* and the bonds are preserved between clusters.

#### 4.2.2 Metal-Oxo

**Figure 6:**
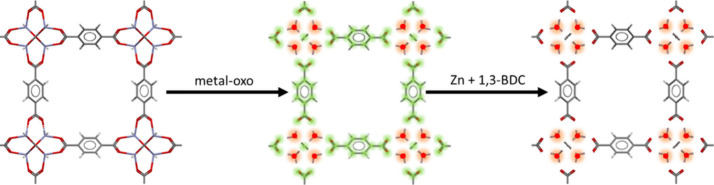
A metal-oxo deconstruction, shown as a schematic diagram, performed on CSD SAHYIK.^66^ In the original structure (left), C (gray), O (red), and Zn (violet). This technique draws many similarities to the single and all node approaches, but with a focus on structure chemistry showing the resultant Zn metals (red) and 1,3-benzenedicarboxylate linkers (green).

The metal-oxo algorithm is a more recently developed technique, created by the Snurr Group to describe MOF chemistry by dividing structures into distinct organic and inorganic building blocks - retaining organic linkers as discrete building blocks (including carboxylate groups).^28^ Compared to the more topologically inclined single and all node algorithms, the metal-oxo approach is a more chemistry focused approach to describe the targeted structure, although it draws some comparisons with the single node approach. The result is achieved by keeping organic linkers intact and therefore it provides alternative information to the other methods. MOF structures are divided into distinct inorganic and organic building blocks via a bond adjacency matrix using a distance cutoff method that adopts the InChI convention of classifying metals and nonmetals. Typically, the inorganic blocks consist of metal-oxo clusters including oxides and bound hydroxide, peroxide and water species with the remaining fragments considered organic building blocks and described as larger nonmetal clusters. These building blocks, represented as SBUs, are characterized by their points of extension, through which they connect to other building blocks in the underlying net.^67^ This distinction between the metal-oxo algorithm, and the single and all node algorithms which consider carboxylates part of the node, can be an important distinction in cases where, for example, five discrete metal atoms are instead represented by a pentametallic SBU.^68^ The metal-oxo approach is shown as a schematic in Figure 6., where it is used to simplify the structure into a complex, metal independent form.

While the metal-oxo method is not typically employed to determine the topology of a structure, due to being primarily developed to offer insight into the constituent metals and linkers of a crystal structure, it is both important and interesting nonetheless to consider alternative approaches to structure simplification.

## 5 MOF Databases and Design Principles

**Figure 7:**
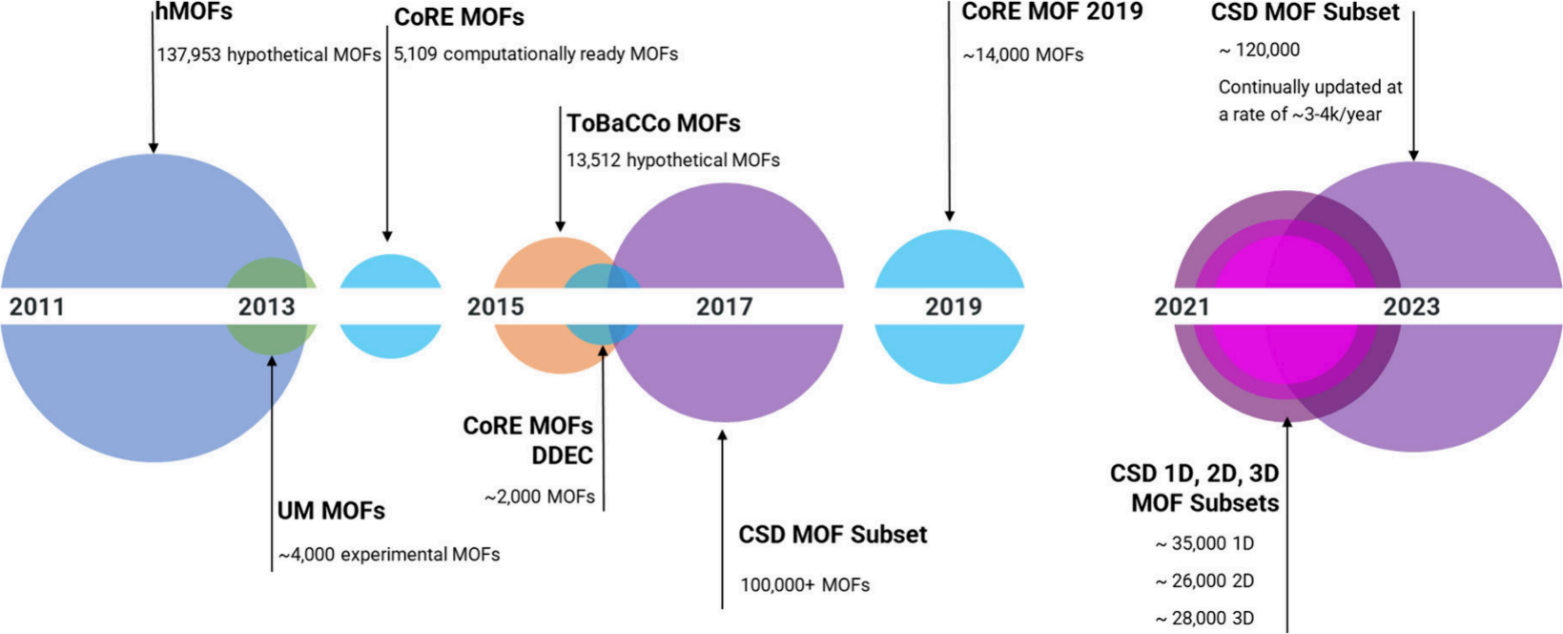
A timeline to show the emergence of selected MOF data sets following the release of the first hypothetical MOF database (hMOF^70^) in 2012. Circle size varies to represent the relative size of the database.

Over the past decade, significant research has been conducted via large-scale high throughput computational screening of structures from various databases containing key information regarding thousands of lab synthesized MOFs or hypothetical materials. Continuous improvement in MOF synthesis practices have led to a greater ability to control key properties of newly created structures, including topology. Over the past 10 years, several databases containing hypothetical and experimental structures have emerged. Figure 7. shows a timeline noting the release date of a handful of selected MOF data sets. For a more comprehensive list of MOF databases, we refer the reader to a recent review by Moghadam et al.^69^

In 2012, Wilmer and colleagues^70^ generated the first database of ca. 130,000 hypothetical MOF structures, containing only a handful of topologies with **pcu** dominance. Later in 2016, Gomez–Gualdron and colleagues^71^ constructed ca. 13,000 structures with 41 predefined nets to enrich MOF topology diversity. The first categorization of large sets of experimental MOF structures began with the creation of the UM MOF database in 2013,^72^ this study was focused on the identification of porous MOFs from the CSD, selected to calculate theoretical limits of H_2_ storage, a study that was completed for ∼4000 MOF compounds out of around 22,000 “computationally ready” candidates. This was closely followed by the development of the Computationally Ready Experimental (CoRE) MOF database in 2014, as part of the Materials Genome Initiative.^73^ Consisting of modified CSD entries, it had been specifically created for use in molecular simulations of gas adsorption. Only 3D structures with pore sizes exceeding 2.4 Å were considered, and over 4,700 porous materials were collected in a computationally ready database. The CoRE MOF database features around 260 different RCSR topologies, plus a select number of EPINET entries. Later, in 2019, the CoRE MOF database saw the completion of an update, increasing the total of porous 3D MOF structures, reported in published literature sources, to 14,000. This new update also added further value to the data set by offering new pore analytics and physical property data alongside the correction and reconstruction of many disordered structures.^74^

In 2017, Moghadam et al.^19^ developed the CSD MOF subset, a searchable database of MOFs that is continually and automatically updated, with additions to the collection every quarter, as new materials are deposited and accepted as part of the CSD. This work created the largest collection of experimentally synthesized MOF-like structures to date (now numbering ca. 120,000 as of April 2023) but was done so using loose definitions to avoid omitting potentially useful or interesting structures, and to allow for an all-encompassing data set that can be further scrutinized by the user depending on their interests. Containing an initial ca. 70,000 1D, 2D, and 3D structures combined, the size of the CSD MOF subset has almost doubled in just seven years. Additional developments to the CSD MOF subset reported in 2020, resulted in the creation of 1D, 2D, and 3D MOF subsets.^20^ While at present there is no option available when browsing CSD structures to easily identify a material’s topology, we are developing methods to perform reliable high-throughput topological allocation on these new subcategories of structures to be included within the CCDC’s database. The CSD 3D MOF subset is an ideal candidate for development into a resource where the inclusion of topological characterisations would become most readily available.

Further to this, we note that the distinction between a set containing all structures and those without disorder is significant in this field where the exact connectivity of atoms is of upmost importance for producing reliable high-throughput topological analysis. It is imperative then, that the first step toward topological identification of any structure found in the CSD MOF subset using these approaches is to determine whether the structure is crystalline, and what level of periodicity it demonstrates. 1D structures, known in the CSD as 1D chains, are not expected to be assigned topology using the techniques outlined in this article. 2D structures, known as 2D sheets, are restricted in their allocation to a limited set of 200 configurations as specified in the RCSR, and due to the limited range and complexity, we expect a significant proportion of these should be identifiable, via the use of software. This distinction into periodic categories enables even the novice crystallographer to quickly determine, by knowing its dimensionality, as to whether an incorrect topological net has been allocated to their structure.

One reason for mismatched topological assignment between dimensionalities could occur due to incorrect bonding determination, for example a 3D structure may be assigned a 2D topology if atom connectivity between 2D layers had not been correctly interpreted–a possible outcome when using automatic bonding assignment software, and one that is particularly prevalent for structures that contain metal–metal bonds. Bond assignments are typically entered by the CSD editorial team with a view to represent the original experimental publication as closely as possible, this is to ensure that the process of assigning bonds is not done entirely on distance - particularly for bridging O or H.

More recent developments in the determination of crystal structures have seen the implementation of machine learning (ML) and specifically the use of neural networks (NN). It was proposed that crystal materials are best represented by multigraph crystal graphs, and the first implementation of Crystal Graphs Neural Networks (CGNN) was made in 2019,^75^ removing the requirement for bond distances and introducing scale-invariant graph coordinators. This has led to the rapid development of other neural network-based approaches ranging from the use of graph neural networks (GNN) to predict material properties,^76^ and analyze the shortfall of lone GNNs to predict material periodicity,^77^ to the use of neural structure fields (NeSF) for the development of autoencoders by representing crystals as continuous fields as opposed to a discrete set of atoms.^78^

**Figure 8:**
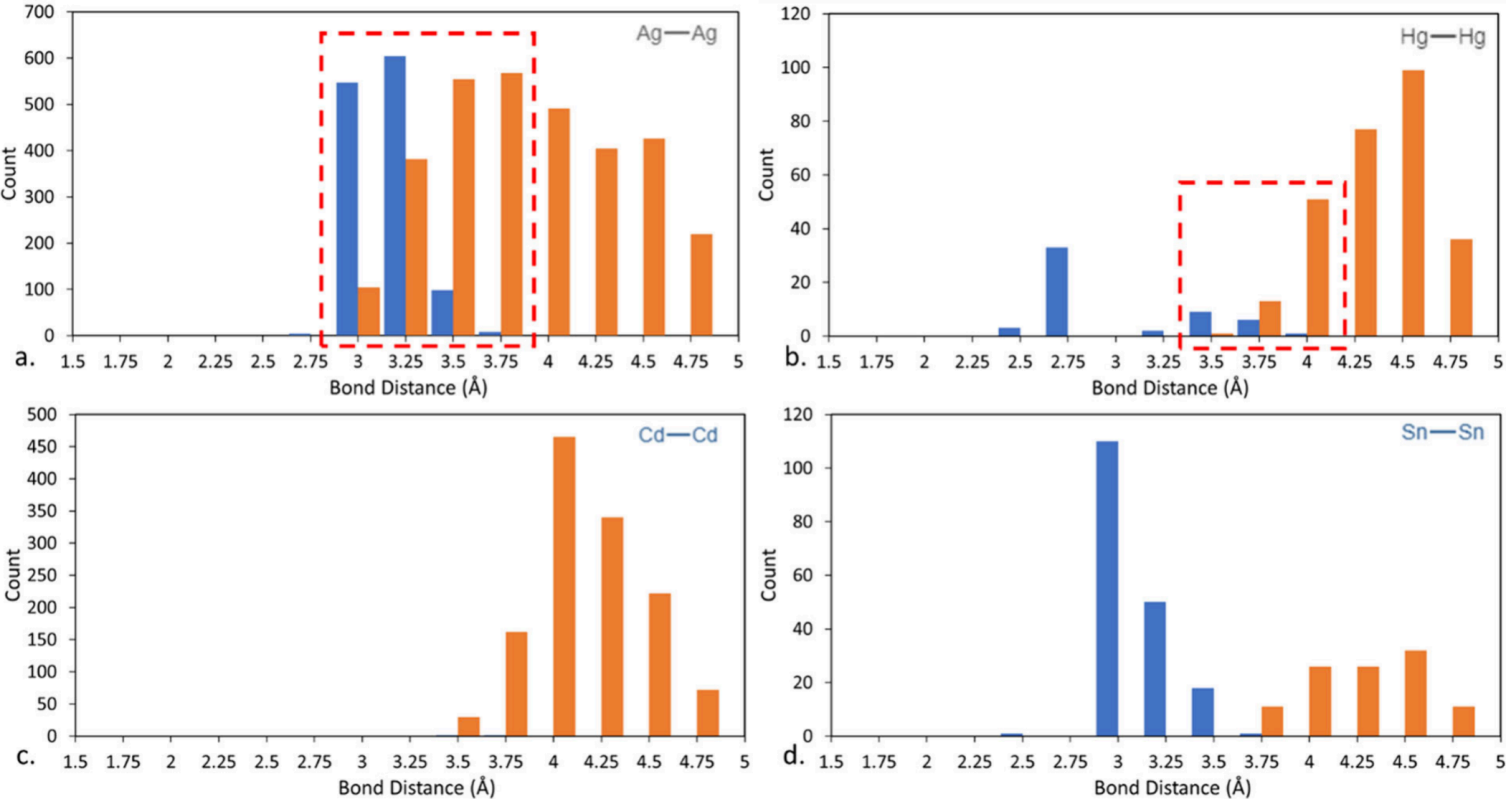
Distribution of selected atom–atom bonded (blue) and nonbonded (orange) contacts (out to VdW+0.0) in the CSD. (a) Ag, (b) Hg, (c) Cd, and (d) Sn. Dashed red boxes suggest contentious atom–atom bonding ranges.

It is important to note that the determination of topology can be dependent on what atom pairs can be considered bonded or nonbonded. Some interpretations may include hydrogen bonding, whereas others may discount metal–metal bonds. At present, the interpretation of bonding can rely on the definitions defined by what a crystallographer considers to be important for their structure e.g. hydrogen bonds may be considered a key part of structure stability. Topological assignment programmes, and indeed MOF databases, can and do have different heuristics when considering atomic bonding. Figure 8 compares the distribution of a variety of metallic (X) X-X bonds and nonbonded interactions within the CSD. In Figure 8a. bonded (blue) and nonbonded interactions (orange) for Ag–Ag fall within a range primarily between 2.7–3.5 Å (represented within the dashed red box). Figures 8b—d show more examples of metals that either have potential atom–atom bond misalignments or metals where this may be of no concern. Hg–Hg shows a similar pattern to Ag where ambiguity may lie (also within the red dashed box) for structures such as the bonded CSD GIZPIP^79^ for interactions between 3.5–4 Å. An apparent lack of data surrounding Cd–Cd bonds here suggests a lack of Cd–Cd based SBUs (highly likely given the bond order calculations for creating Cd–Cd bonds^80^) with only 6 nondisordered MOFs containing a Cd–Cd bond, and last the Sn–Sn data shows an example of clear delineation at approximately 3.75 Å between bonded and nonbonded contacts. In the dashed red regions, we expect to see examples of both bonded and nonbonded layers in 3D Ag and Hg containing structures, a highly important detail when we consider the use of autobonding software in the topological assignment process, but conversely structures such as Sn–Sn would be ideal candidates for investigation where the use of automatic-bond assignment software could be considered less troublesome. Subsequently, the 6 Cd–Cd bonded structures identified here were investigated and manually corrected as a result of this study. While we have mentioned only a select few examples here, metallic bonding data is available for all structures in the CSD.

**Figure 9:**
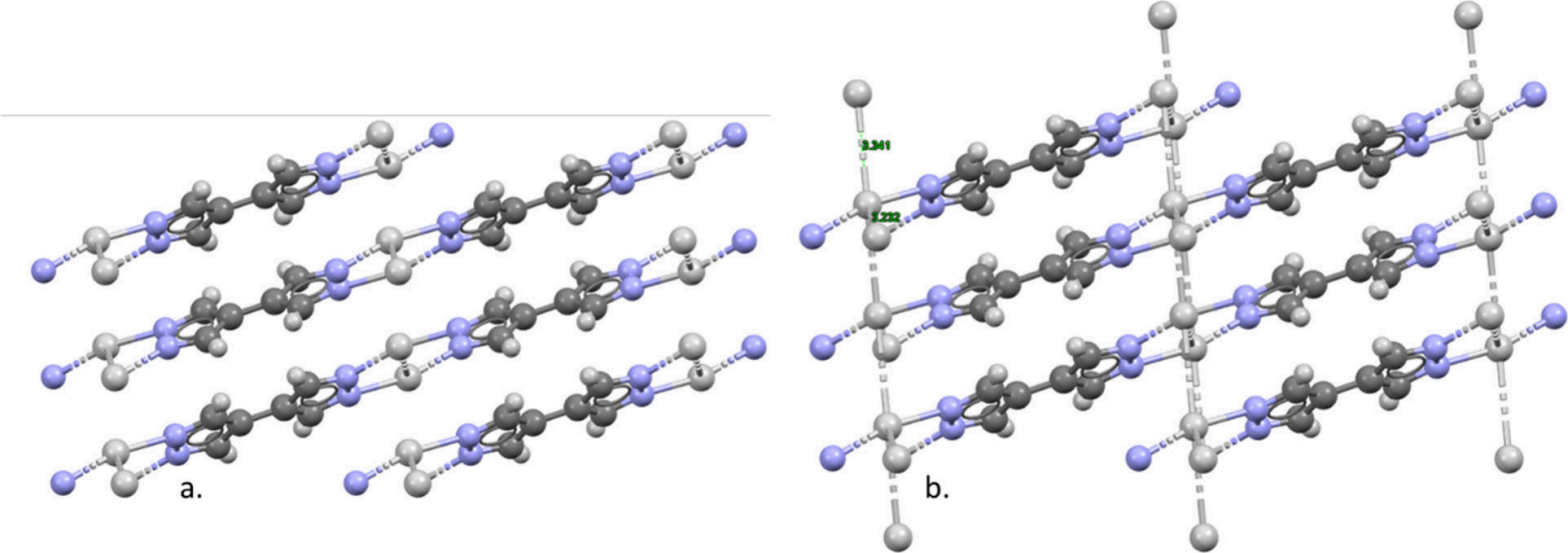
Atomic representations of CSD ZEHMOQ showing (a) the original structure set at a 3.32 Å Ag–Ag bond distance limit and (b) an automodified version with a 3.35 Å Ag–Ag bond distance limit, where the connectivity has been calculated using automatic bond assignment tools within CSD Mercury.

To highlight the importance of bond assignment in determining structure dimensionality, let us investigate an example. Figure 9 shows CSD’s ZEHMOQ,^81^ a 2D MOF containing Ag–Ag bonding at 3.32 Å, however, extending the bonding limit just slightly to 3.35 Å (which could be considered a possible bonded or nonbonded distance) transforms the 2D sheets into a single 3D crystal structure. Here, we note that taking atom connectivity data directly from the CSD before modification offers a more chemically aware insight into the structure of a crystal, as determined by the experimentalists themselves when depositing structure information, rather than risking miscalculation by automatic bond assignment software with algorithms deciding bonding based on atomic distance.

We would recommend that when attempting to assign topology to a structure that the original chemical bonding is considered (wherever possible), as opposed to removing/omitting the existing bonding data and attempting to reassign it using additional software such as OpenBabel.^82^ Therefore, while most topological characterization software is packaged with some form of bonding assignment tool to calculate atomic bonding for imported CIFs, we recommend inclusion of the CSD’s atomic bonding data in all generated CIFs. Although this is available for structures obtained through the CSD’s Python API, typical CIFs do not contain atom–atom bonding information. Further to this, even if the bonding data is present, it is not always possible to upload a CIF to these software packages and retain the relevant CSD bonding data as the only option available may be to recalculate bond types and distances, and while these may be manually edited later, structures requiring manual bond modification may restrict the capability for high-throughput calculations.

## 6 Topological Characterization Software

### 6.1 Introduction

At present, a handful of topological identification tools exist, aside from the painstakingly slow and perhaps unreliable method of performing manual structure-net matching. Historically, the Java based Systre^51^ program has been used to identify RCSR topologies before the introduction of new software ToposPro in 2014. Released in 2003, Systre, part of the Gavrog project has been used extensively to characterize underlying MOF structures, but its applications are limited to RCSR nets as of 2013, and there are 13 known RCSR nets it does not currently recognize. For 10 years, Systre was the go-to program for topological assignment of MOFs, and its development stimulated the development of newer and more powerful approaches.

Now, the most well established and frequently cited package is ToposPro.^26^ The developers at Samara continue to maintain this software, have published many video guides for inexperienced users, provide in person training at summer schools and conferences, and even offer a topological identification service for a fee. A more recent development, which has seen some updates this year for use in high-throughput topological assignment approaches is MOFid.^28^ MOFid has been used as a topological identification software for the CoRE MOF database so that topology can be searched for within the data set, but its primary use is focused on obtaining unique identifiers for MOF linkers. Finally, and most recently published is the CrystalNets package,^29^ and although this software has been published and is available, not enough opportunity has been given since its release in 2022 to judge the uptake of this approach within the community, aside from a small number of interesting citations. These software packages have all been built using different programming languages and offer the user multiple approaches to verify the output of their structure’s topological identification.

### 6.2 ToposPro and TopCryst

ToposPro is a licensed downloadable program, available for Windows users, that is frequently maintained and updated, with the latest version 5.5.2.2 available at https://topospro.com/, that can be activated using a free license provided for academic users. An entirely automated version can be implemented for single structure analysis without requiring any installation by uploading a CIF online at https://www.topcryst.com. The topology of a single structure can also be quickly obtained by searching the TTD database. This service is restricted to 10 uploads per user per day which could limit its use for high-throughput applications. An added, and useful, feature of this online tool allows a user to search for any 3-letter RCSR topological representation and view this in a JSmol window at various dimensions of unit cell, with several example structures from the CSD also shown in a table below the topological search. This is not a complete open-source online database of structures as the free version does not allow the user to download the CSD refcodes of any specific topology, but instead offers five random examples of structures which meet the criteria of the searched topology and notes the technique through which they were obtained. We also note that there is no.

**Figure 10:**
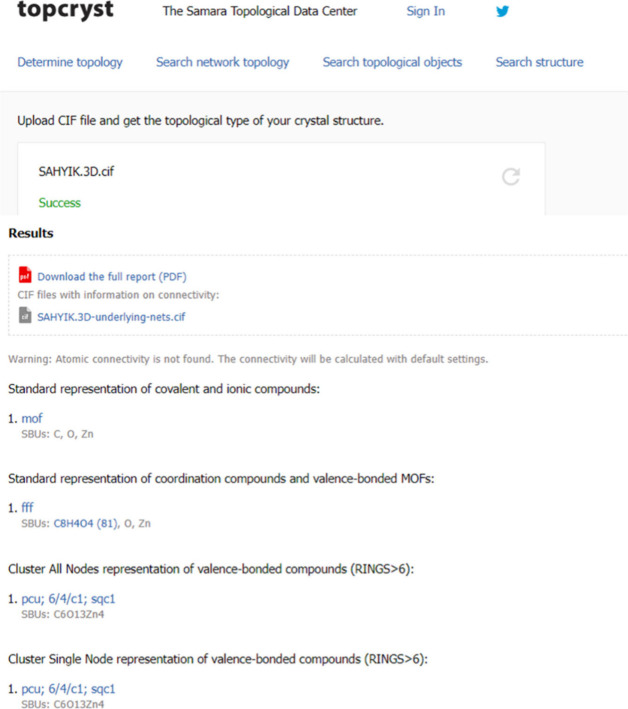
A snapshot of the online interface of the TopCryst web topology service used for the automatic deconstruction of CSD SAHYIK. The original CIF was modified with the use of CSD’s Python API solvent removal script.

An example is shown in Figure 10 using CSD SAHYIK, more commonly known as MOF-5, with the TopCryst online interface after uploading a CIF file where the unbound solvents have been removed. Here we can see the allocation of three distinct RCSR topologies, **mof**, **fff**, and **pcu,** with a clear indication of the methods used to obtain each underlying net.

With regards to the software itself, ToposPro is a program package for comprehensive analysis of geometric and topological properties of periodic structures such as, but not limited to, MOFs. The techniques contained within can be applied to almost any structure of a chemical nature. It has been developed to process large crystallographic data samples and correlate structure property parameters. The principles behind this software package aim to achieve a human independent crystallographic data processing tool which approaches materials that have a variety of complexity levels with universal algorithms in contrast to traditional crystallochemical visual analysis. The aim of separating structures using universal algorithms is an effort to avoid the difficult nature of topological assignment and offer consistent topological representation of structures by minimizing any errors. This method is known as the Domains algorithm which uses atomic Voronoi polyhedra as geometrical parameters of atoms and bonds.^26^

All methods contained within ToposPro can be divided into geometric or topological groups, respectively. The first group is concerned with routine geometric calculations and crystal structure visualization, and the second contains the procedures required for studying connectivity of the whole crystal environment. A database is created upon the importation of a CIF, and bonding must be assigned to structures added to the database before topological assignment can occur. This is performed using the AutoCN program, the details of which can be found in the ToposPro manual. It has been tested on thousands of structures from the CSD and has showed good agreement with chemical models.^64,84^ For structure deconstruction, the use of cluster representation is possible in three different ways, using the chemistry mapping single node, the geometry mapping all node, and the tertiary building unit (TBU) cluster mode. There is the additional possibility, which is applicable to all structures, called the ToposPro standard, or standard representation, mode. It should be noted that this is not always the most descriptive method, and typically more information can be obtained using other approaches. Additional features of ToposPro include the ability to detect duplication of structures, investigate entanglements and interpenetration, and the modification of structure bonding following the use of AutoCN. The software is noted for its high accuracy when implemented on suitable structures following the AutoCN stage.

The limitations of the software include the application of the program on large data sets, and while it is possible to run continuous calculations on tens of structures at once, the nature of the program restricts the use of true high throughput operation. The ToposPro package is best suited to investigating individual structures on a case-by-case basis, and when using this approach it is a powerful tool for topological assignment, particularly when focused on rod-like MOFs as other packages struggle to handle these difficult to interpret materials.

### 6.3 MOFid and web-mofid

MOFid^28^ is a freeware Github hosted identification software available at https://github.com/snurr-group/mofid. The primary MOFid package can be downloaded and installed using a make file directly into a virtual environment. Any CIF located in an accessible directory can be parsed using the cif2mofid function of the MOFid program for topological analysis directly from the command window within a python environment. It is worth noting that the software package has had a larger focus on the identification of linkers than topology and is primarily designed to offer insights into MOF building blocks by assigning the linkers with unique identities to improve the cross referencing of linkers between MOF structures that share some of the same building blocks.

**Figure 11:**
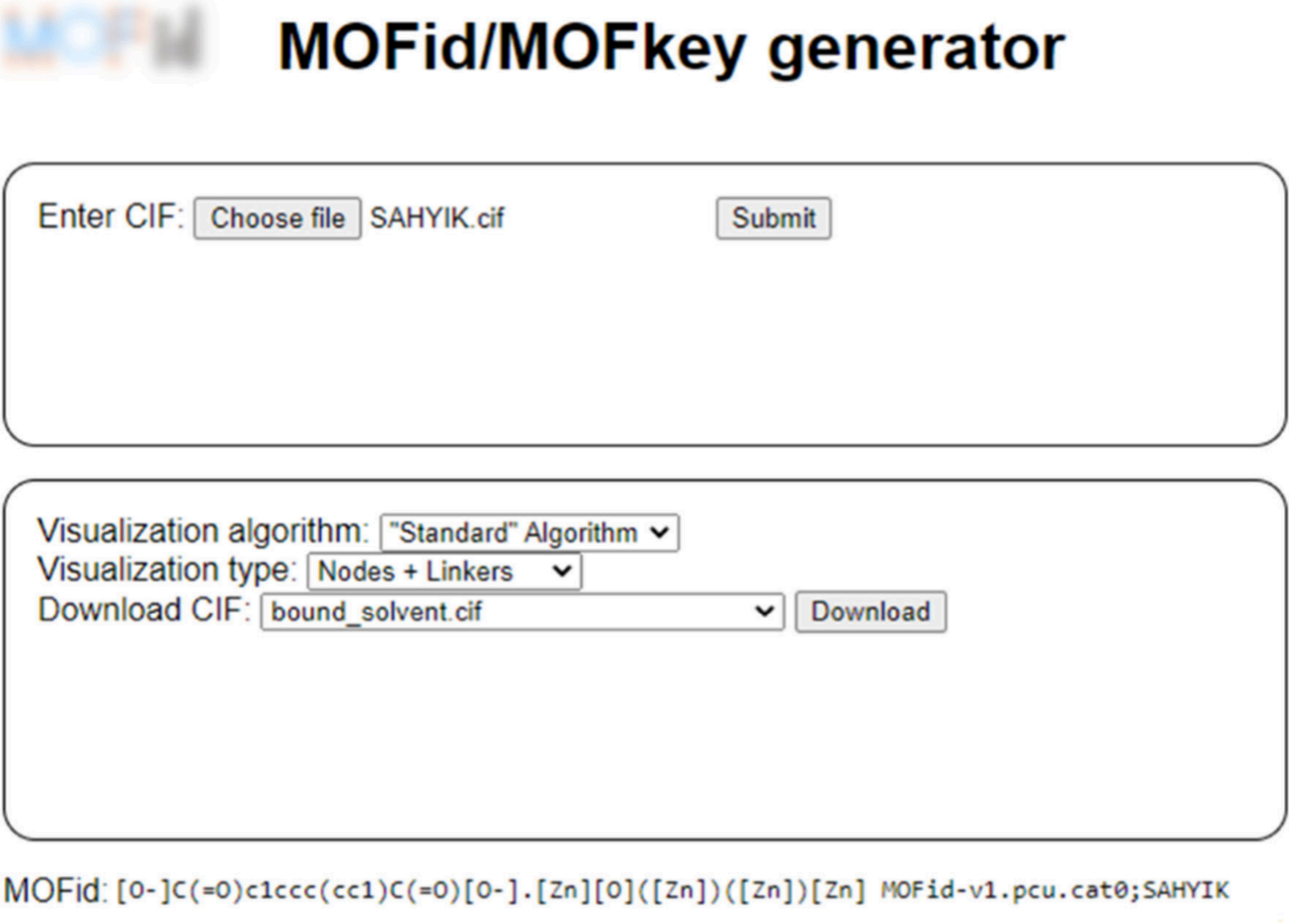
A snapshot of the online interface of MOFid’s web structure identification and topology tool performing a structure simplification on CSD SAHYIK by uploading the raw CIF.

Similarly to TopCryst there is a single structure web-based analysis feature into which CIF files can be uploaded for topological analysis as well as deconstruction into individual building blocks followed by the allocation of identifiers, this can be found at https://snurr-group.github.io/web-mofid/. Not only does MOFid return a topology parameter, but it also returns a MOFid or MOFkey string. A MOFid is based upon SMILES strings and takes the form of inorganic building block, organic building block, format, topology code, catenation, comment. A typical example of a MOFid for Cu-BTC would be [Cu][Cu].[O-]C(=o)c1 cm^3^(cc(c1)C(=O)[O-])C(=O)[O-] MOFid.tbo.cat0;Cu-BTC. This can be pasted into any software package that recognizes SMILES, such as ChemDraw, and it should render for visualization. The alternative output is the MOFkey which takes a similar form as above except with the catenation and comments no longer present, and the organic building blocks now represented by a unique alphabetized code. The same Cu-BTC structure as above has the MOFkey as follows: Cu.QMKYBPDZANOJGF.MOFkey-v1.tbo. Figure 11 shows the output of CSD SAHYIK uploaded in CIF format to the web interface of MOFid, displaying the options for algorithm visualization in a drop-down box, and the corresponding MOFid text-string below.

A final web-based feature is the CoRE MOF database search tool^28^ which allows a user to search over 15,000 MOFs by SMILES/SMARTS, topology, or catenation. A simple text-based search in this data set for **pcu** reveals 749 MOFs and their SMILES string, catenation, and where applicable their CSD refcode. If a user’s chosen refcode matches a structure in CoRE MOF, there is no requirement for the user to rerun any structures found in the database to obtain these parameters.

The MOFid Github package also contains shell scripts to run a directory of CIFs on a high-performance computing cluster, and it is possible to process a folder containing thousands of MOFs, provided that the input files are suitable for the software. Bonding is assigned using the open source OpenBabel chemical toolbox that was designed for use with molecular modeling, chemistry, solid-state materials, or related applications.^82^ OpenBabel can implement a wide range of cheminformatics algorithms including bond order perception, once the unit cell information is extracted from a CIF file.

Simplification is performed by the metal-oxo, single node, and all node algorithms with the output of each technique available to visualize via the dropdown box. This feature is particularly useful to compare the different methods, although the output string containing the topology reports only one underlying net even if several have been detected.

The simplified net is exported to Java based net matching program Systre,^51^ where the RCSR nets are preloaded, and the new simplified net is matched to one of the existing configurations within this data set. The use of this program within MOFid is key to the topological identification stage and the speed at which this matching is performed can be a limiting factor in the high-throughput use of this software when compared with CrystalNets which does not require the use of Systre.

It is possible when using the MOFid python package to modify the output desired by the user by editing a few simple lines of Python code. By performing this modification, a user can report topology based on whatever criteria they so choose, and for example might only be interested in structures where the topology obtained via the single and all node algorithms are the same. It would be equally as simple to report topology for only structures where the output between the two techniques is different, or for all three methods contained within this software.

### 6.4 CrystalNets.jl and CrystalNets

CrystalNets.jl^29^ is an open-source Julia based software package hosted in Github, that can be obtained from https://github.com/coudertlab/CrystalNets.jl. The installation can be performed quickly and easily after opening Julia, by entering the package manager and adding CrystalNets, and the integration of the program within a Python environment can be enabled with relative ease. It is possible to install the package as an executable for a handful of structures, but for high-throughput approaches the use of CrystalNets as a Julia module is recommended. This software is specifically designed for the automatic detection and identification of underlying topological nets of crystalline materials, and the input format can follow any file type that is recognized by chemfiles.^85^

Upon installation, there are a variety of settings available to the user, the most basic of these includes the ability to select the deconstruction algorithm used, whether to use the bonds that are input in the file or to guess them, and the type of structure that is being investigated. In this package the standard, all node, and single node approaches are available, so for example, it is possible to select MOFs, deconstructed using the all node algorithm, with the guess function enabled for bonding if they were not included in the original input file, or auto if some files contain bonds and others do not. This feature is particularly useful for defining the topology of MOFs where there are bonding parameters contained within the input file should the CIF have been taken directly from the CSD with care taken to ensure that the bond lengths have been retained. There is also the availability of a MOF option which modifies the approach to enable the detection of organic and inorganic clusters, allowing them to be subdivided using either all node or single node algorithms to identify the underlying nets. Other choices for this parameter also include Zeolite, Cluster, Auto, and Guess. The CrystalNets manual is a good accompanying resource that contains all the available options for each function and further explanations surrounding exactly what each of the changes to these input parameters makes to the process.

The use of a Julia module allows for some extremely fast structure deconstruction compared to the other methods available, and this is amplified by the availability of a multithreaded implementation for a large set of structures. The CrystalNets program is orders of magnitude faster on a typical laptop running a few threads compared to the automated and high throughput MOFid approach even when it is performed on several nodes of a high-performance computing cluster, and of course quicker still than the more user dependent ToposPro approach that requires much more user interaction than the other techniques. CrystalNets has the power to perform topological identification on tens of thousands of MOF and MOF-like structures with notable reliability. In a recent study by Burner et al.^86^ this software was used to identify the topology of 72,257 MOFs, for a new database ARC-MOF, with a match to file name 93% of the time and at a rate that can vastly outperform a competent and experienced researcher investigating a single structure in ToposPro. The entire database of ARC-MOF could be assessed within a single afternoon on a regular computer using a multithreading approach.^86^

**Figure 12:**
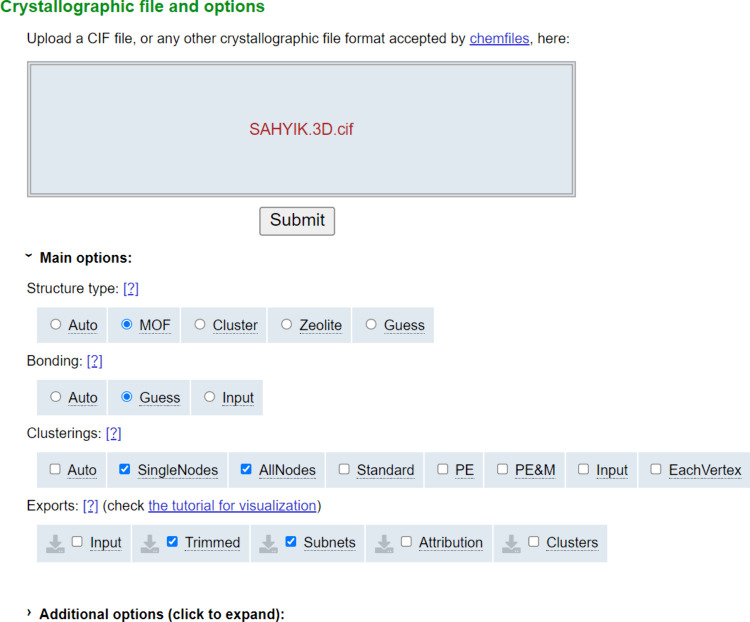
A snapshot of the online interface of MOFid’s web structure identification and topology tool, showing the options available for each uploaded CIF file.

In addition to the Julia module there is also an online web interface which allows for the upload of CIF files, with a more user-friendly process for topological identification of individual structures than running them in the Julia interface. The available options using the newly released online version of the CrystalNets software can be seen in Figure 12.

The online version of CrystalNets is not dissimilar to the online interfaces of MOFid or TopCryst, boasting a visualization tool that shows a simplified net overlaid on the original structure. One major difference is the ability to select the structure style and bonding settings before the structure is uploaded. This is useful for a user who may know specifically which algorithm to select, whereas the reporting of all potential nets by TopCryst via each technique is more suited to a more inexperienced crystallographer.

### 6.5 Guidance and Limitations

One major limitation of these high-throughput automated approaches for topological assignment of crystalline materials via the medium of CIFs is the lack of verifiability of results returned using these software packages given the subjective nature of topology assignment, something which is only addressed using a manual topological assignment tool. However, the possibility to analyze a prospective structure within several different programmes allows for more certainty surrounding the identification process than using a single approach, particularly when considering the similarity in deconstruction algorithms used across these platforms. We recommend that topological analysis is performed using at least two software packages, running the same algorithm, to verify the results. Should the case arise that the two results disagree then further, more detailed investigation must take place. Further investigation could include checking the periodicity of a structure, in cases where different software suggest 2D or 3D structures. One could also look at bonding data from each software and check for potential differences or errors between input CIFs ensuring that the same parameters e.g. deconstruction method or type of structure are selected to minimize such discrepancies.

We must consider that some key differences between these software packages exist, the most notable being the technique used to assigned bonds between atoms in nonbonded CIFs. These bonding approaches, despite their apparent similarity contain subtle differences in their approach and it is these subtle differences that can create major changes in the outcome of topological assignment software. To check that topology for a large set of structures has not been incorrectly assigned, it is possible to cross reference structure refcodes with the CSD’s 1D, 2D, and 3D structure subset, and the resultant topology with the RCSR’s 1-periodic, 2-periodic, and 3-periodic net database, however some errors may persist.

## 7 Recent Developments

In recent years, many groups around the world have implemented ToposPro to identify the topology of individual structures, or larger sets of crystal data, and used this information alongside other properties to create data sets for MOF and MOF like materials. In a recent study by Cheng et al.,^87^ Topos software was used for the topological classification of coordination polymers which were generated in the exploration of H_2_pdba, an adaptable linker. It was used to assemble a diversity of new Mn, Co, Ni, and Cu coordination polymers into 2D metal–organic layers and 3D MOFs which disclosed several types of topologies including **sql**, **hcb**, and **tfk**. There are many examples of the implementation of Topos for structure analysis within the community and these can be found within the 2000+ citations of the ToposPro software package, although not all of these publications are exclusively MOF related. It is imperative in this review that we should include the introduction of the TopCryst online package^88^ which was made available for use only in March 2022, followed very quickly by that of the CrystalNets web interface that came online just six months later in October 2022. The TopCryst service has already been cited several times in significant journal publications within the first six months of its release.

There have also been several examples of recent implementations of MOFid to explore the importance of structure topology. One primary example is the recent publication of the Automated Reticular Framework (RF) Discovery platform by Pollice et al.^89^ in 2021 where they implement data obtained using the tools published in MOFid for a data-driven strategy focused on accelerated materials design.^89^ Knowing the physically feasible topologies for structures based on chosen linkers has also been useful for bottom-up MOF building approaches where the topologies and linkers of previously synthesized MOFs had been extracted from the CoRE MOF database using MOFid.^45^

In another study, MOFid was used to identify Cu paddlewheel MOFs from a set of 1172 nondisordered MOFs to investigate structural collapse during activation.^90^ Once these structures were gathered it was possible to perform high-throughput computational analysis to investigate the effect of various mechanical properties.

Lastly, the CrystalNets publication, despite its recent publication, has already received several citations from studies focused on the topological identification of MOF structures. It was first used in print to characterize the topology of ∼100 Zr-oxide MOFs, before it was then applied to much larger sets of data by Burner et al. on a group of approximately 72,000 MOFs that included previously known topologies.^86,91^ Later, Glasby et al. ran CrystalNets on ca. 28,000 experimental MOFs from the CSD 3D MOF subset for the first time during the development of the DigiMOF database.^25^ Mournio et al. also used CrystalNets to characterize the topology of over 300 COFs as prospective candidates for photocatalysis, showing that the use of this software is not limited to MOFs alone.^92^

## 8 Conclusions and Perpsective

The availability of these software packages shows that topological characterization of crystal structures is important, not only to MOF researchers but also to those interested in COFs, Zeolites, and other crystals that form periodic networks in their atomic structure. MOF synthesis can play a major role in topological determination as different conditions lead to the formation of topologically different structures, influencing not only the resultant mechanical stability but also the pore shape and sizes of a crystal depending on the SBUs and linker types that have been selected for their synthesis.

The choice of topological assignment software is highly likely to depend on the requirements of the individual study, as each different tool has its own strengths and limitations. CrystalNets is more notable for its speed and its ability to read in atom bonding information, but it does not offer the same chemical structure insights as MOFid for example, and its choice of topological representations is limited compared with ToposPro. However, ToposPro has an advantage in that any structure can be manually modified during the deconstruction process increasing accuracy when used by experienced crystallographers compared to fully automated methods.

A notable limitation of all software approaches is when comparison between single node and all node topology allocation differ from each other. Following IUPAC guidelines as outlined in this article, any cases where a different net is reported the result should be designated as “the xxx-derived net yyy”, something we note is seldom seen. A simple change in the software output to reflect this might help researchers to ensure they are reporting in line with the guidelines.

Lastly, we reiterate that to date there is not yet a complete, freely available database of MOFs that contains the relevant RCSR or other topology type for all structures that has been proven and adequately verified. The introduction of resources such as the QMOF database,^93^ which contains over 20,000 MOFs and their quantum-chemical properties serves as an example of the importance of publishing key data to limit the need to repeat computational calculations between research groups. Once a database of MOF topologies has been properly curated and confirmed it can prevent the need for repetition. While the topologies reported in the CoRE MOF database has been a good start, there are still improvements to be made.

The CSD is an ideal target for a database that could include topological information published during deposition given its manual curation, continuous quarterly updates, and extensive searching tools. While we note here that the CSD system itself is not freely available, individual structures are through the CCDC’s access structures service, and should the relevant topology be contained within a deposited CIF then that information would become freely available, as the individual deposited CIFs can be downloaded from the respective entries.
